# Spontaneous Spinal Epidural Hematoma Associated With Apixaban Therapy: A Report of two Cases

**DOI:** 10.7759/cureus.11446

**Published:** 2020-11-11

**Authors:** Frank M Mezzacappa, Daniel Surdell, William Thorell

**Affiliations:** 1 Neurological Surgery, University of Nebraska Medical Center, Omaha, USA

**Keywords:** spontaneous spinal epidural hematoma, apixaban, case report, eliquis

## Abstract

Spontaneous spinal epidural hematoma (SSEH) is a rare clinical entity that can result in severe neurological deficit and warrants emergent neurosurgical evaluation and management. The exact etiology of this entity remains unknown, but certain risk factors exist, including the use of anticoagulant medications. There are few published reports of the association of SSEH with direct factor Xa inhibitors. We aimed to present 2 cases of SSEH in patients on chronic apixaban therapy. To the best of our knowledge, there is only 1 other report of SSEH in the setting of apixaban therapy. A comparison between the cases suggests the importance of rapid recognition and management of SSEH in order to achieve favorable neurological outcomes.

## Introduction

Spontaneous spinal epidural hematoma (SSEH) is a rare clinical entity that can result in severe neurological deficit [1,2]. An exact etiology for this phenomenon remains unknown to this point, but may be the result of rupture of the epidural venous plexus [3]. Early identification is important in order to improve the chance of a favorable neurological outcome. Knowledge of risk factors, such as anticoagulation use, can assist with prompt recognition of the condition [1]. The novel oral anticoagulant medications are increasing in use due their ease of use and efficacy, but there are few reports of SSEH associated with these medications to this point. This group of medications includes the direct factor Xa inhibitors such as apixaban, betrixaban, darexaban, edoxaban, otamixaban, and rivaroxaban. To the best of our knowledge there is only one previously published case report of SSEH associated with apixaban therapy [4]. We aimed to describe our experience with two cases of SSEH in the setting of chronic apixaban therapy in order to raise clinical suspicion and promote rapid diagnosis and management. 

## Case presentation

Case 1

**Figure 1:**
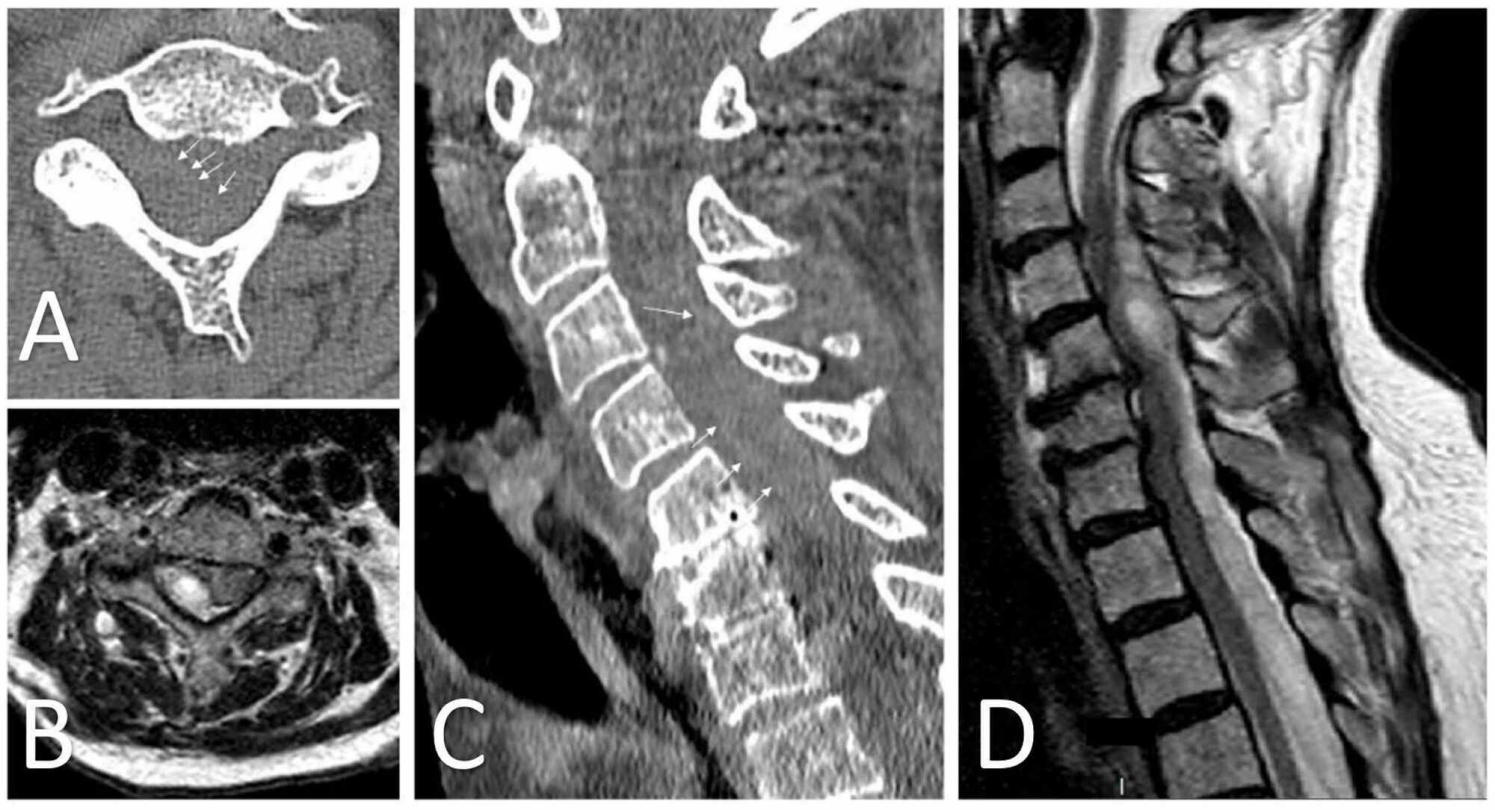
Acute epidural hematoma spanning from C2-T3 Axial (A) CT and (B) T2-weighted MRI demonstrating evidence of acute epidural fluid collection with central canal stenosis and spinal cord compression. Sagittal (C) CT and (D) T2-weighted MRI in the same patient. Hemorrhage is eccentric to the right, consistent with the patient’s more severe right-sided symptoms on presentation. White arrows demonstrate the hemorrhage border with the spinal cord on CT.

This is a 73-year-old female with a known past medical history significant for protein C deficiency on chronic apixaban therapy, polycystic kidney disease status-post kidney transplantation, congestive heart failure, coronary artery disease, gastroesophageal reflux disease, hypertension, and pulmonary hypertension. She presented to the emergency department after developing sudden, severe pain in her neck associated with progressive weakness and sensory changes. Her pain began suddenly while walking to her bathroom. She specifically denied any trauma at the time pain began or in the preceding 24 hours. Subsequently, she developed weakness in the right arm and leg that was associated with progressing paresthesias in the right leg. Hence, her husband brought her to the emergency department at our institution for evaluation, which was approximately 2 hours after initial onset of symptoms. A CT of the cervical spine without contrast was obtained and demonstrated an acute hematoma in the spinal canal (Figure 1A, 1C). Our service was consulted at that time for further evaluation and she was given prothrombin complex concentrate for apixaban reversal. An MRI of the cervicothoracic spine with and without contrast was obtained for further characterization after our initial exam, which demonstrated an epidural hematoma causing spinal cord compression and spanning from approximately the C2 level through the upper thoracic spine (Figure 1B, 1D). This was negative for evidence of an underlying lesion as a cause for hemorrhage. 

Our initial exam was significant for right-sided weakness as follows: 2/5 strength for shoulder shrug and shoulder abduction; 3/5 for elbow flexion and extension; 1/5 for wrist flexion, wrist extension, and handgrip; 2/5 strength for hip flexion, knee flexion and extension; and 4/5 strength for ankle dorsiflexion. The patient was also diffusely 4/5 for strength in all muscle groups tested in the left upper and lower extremities. Reduced sensation to light touch was noted in all dermatomal distributions of the right lower extremity. Deep tendon reflexes were 1+ bilaterally for knee jerk. Her exam was otherwise unremarkable. The initial blood pressure recording was 179/88, so the patient was started on a titratable nicardipine infusion for management with a goal systolic blood pressure less than 140. Laboratory work-up demonstrated PT 10.7 seconds (nl. range 10.1-14.2 seconds), INR 0.9 (0.9-1.1), and PTT 28.7 seconds (24.0-26.0 seconds). Additionally, hemoglobin was 10.5 g/dL (11.0-15.1 g/dL), hematocrit 32.9% (33.1-44.5%), and platelet count 116x10^3^/uL (15-400x10^3^/uL). Due to the clinical exam and imaging findings, the patient was taken directly to the operating room from the emergency department for emergent evacuation of the epidural hematoma. A partial laminectomy of C2 with C3-T2 laminectomies and a right hemilaminectomy at T3 was performed. Acute hemorrhage was noted in the epidural space and this was evacuated successfully. No obvious source of bleeding was identified intraoperatively. The procedure was performed without complication and was tolerated well by the patient. The final pathological analysis of evacuated material was consistent with acute hemorrhage. The patient was started on enoxaparin 40 mg daily on postoperative day number 2 for deep venous thrombosis prophylaxis. Her apixaban therapy was restarted on postoperative day number 14, which was deemed the most appropriate timing based on multi-disciplinary discussion of the limited currently available data in the literature. She was ultimately discharged to a rehabilitation unit after recovery in the hospital. A complete neurological recovery other than residual 4-/5 weakness for right triceps extension was noted at a 2-month follow-up appointment. 

Case 2

**Figure 2:**
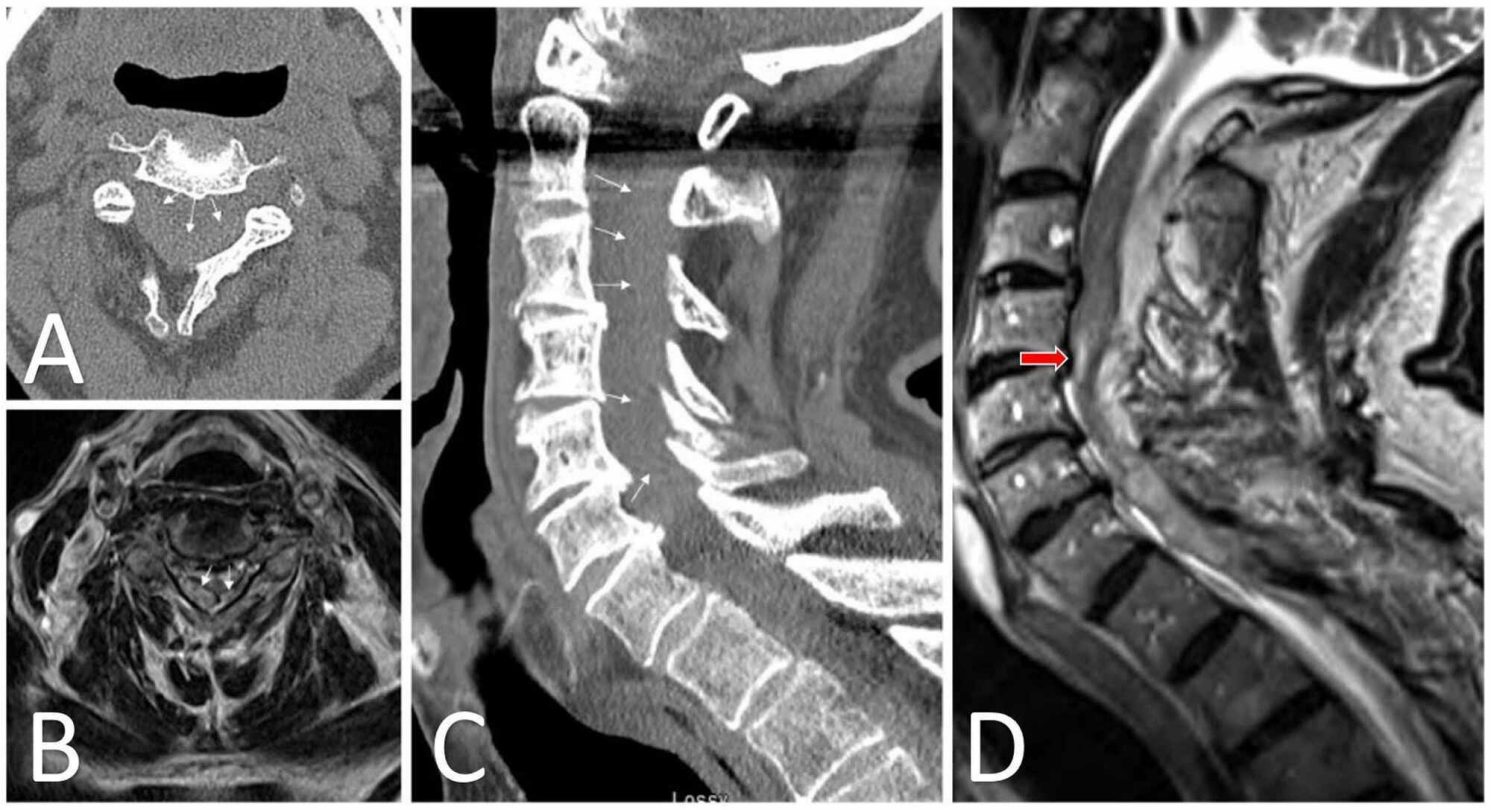
Acute epidural hematoma spanning from C2-T1 Axial (A) CT and (B) T2-weighted MRI demonstrating evidence of epidural fluid collection with central canal stenosis and spinal cord compression. Sagittal (C) CT and (D) T2-weighted MRI in the same patient. White arrows demonstrate the hemorrhage border with the spinal cord. Spinal cord signal change is evident adjacent to the C4-5 disc space (red arrow, D).

This is an 84 year-old male with a known past medical history significant for atrial fibrillation on chronic apixaban therapy, benign prostatic hyperplasia, coronary artery disease, right basal ganglia hemorrhage 2 years prior to current presentation without residual deficit, hypertension, IgA deficiency, right iliac artery aneurysm, and obstructive sleep apnea. The patient reported that he got up from bed at approximately 0500 to use the bathroom and felt a sudden pop in his right neck and shoulder, which quickly progressed to bilateral lower extremity weakness and difficulty remaining upright. He was able to lie back in bed and call emergency medical services, who brought the patient to an outside hospital emergency department for evaluation. CT of the neurological axis was performed and demonstrated an acute hemorrhage spanning from approximately C2-T1 within the spinal canal (Figure 2A, 2C). He was managed expectantly at that time. The patient’s weakness continued to progress while at the outside hospital, so an MRI of the head and cervical spine was obtained in the afternoon, which re-demonstrated the epidural hematoma with severe spinal cord impingement and evidence of cord signal change (Figure 2B, 2D). At that time, our institution was contacted for transfer for further evaluation and higher level of care. He was accepted for transfer, ultimately arriving at our facility at 2030. He was given prothrombin complex concentrate upon arrival for reversal of apixaban. 

Our initial exam was as follows: Right-sided motor strength testing: 4-/5 for elbow flexion; 1/5 for elbow extension; 0/5 for wrist flexion, wrist extension, and handgrip; 0/5 for hip flexion, knee flexion and extension, ankle dorsiflexion and plantarflexion. Left-sided motor strength testing: 3/5 for elbow flexion; 1/5 for elbow extension; 0/5 for wrist flexion, wrist extension, and handgrip; 0/5 for hip flexion, knee flexion and extension, ankle dorsiflexion and plantarflexion. Light touch and pinprick sensations were absent in all distributions below the C6 dermatome bilaterally. Biceps reflexes were 2+ bilaterally, but triceps, brachioradialis, and patellar reflexes were 0 bilaterally. The initial blood pressure recording was 191/90, so the patient was started on a titratable nicardipine infusion for management. Laboratory work-up demonstrated PT 14.0 seconds (nl. range 10.1-14.2 seconds), INR 1.2 (0.9-1.1), and PTT 26.8 seconds (24.0-26.0 seconds). Additionally, hemoglobin was 10.3 g/dL (11.0-15.1 g/dL), hematocrit 29.8% (33.1-44.5%), and platelet count 138x10^3^/uL (15-400x10^3^/uL). We elected to take the patient to the operating room emergently without further imaging due to his severe and worsening deficits. C3-7 laminectomies with partial C2 and T1 laminectomies were performed. An acute epidural hemorrhage spanning from approximately the C3-7 levels was identified and evacuated. No obvious source of bleeding was identified intraoperatively. The procedure was performed without complication and the patient was transferred back to the intensive care unit for further treatment and monitoring. Final pathological analysis of the surgical specimen was consistent with acute hemorrhage. Enoxaparin 40 mg daily for deep venous thrombosis prevention was started on postoperative day number 5 after surgical drain removal. The patient's postoperative course was complicated by aspiration with respiratory failure requiring tracheostomy and poor nutrition requiring percutaneous gastrostomy tube placement. We engaged in multi-disciplinary discussions regarding restarting apixaban for the patient's paroxysmal atrial fibrillation in the setting of SSEH. The risk of re-hemorrhage after restarting this medication was felt to outweigh the small benefit on ischemic stroke risk reduction in this clinical scenario, so apixaban was not restarted at the time of last follow-up with neurosurgery. Unfortunately, the patient did not experience improvement in his neurological function after surgical intervention, which was attributed to complete spinal cord injury secondary to long-duration, severe compression from the epidural hematoma. 

## Discussion

Here we present 2 cases of SSEH in the cervical spine in patients on chronic anticoagulation therapy. Both patients underwent surgical evacuation shortly after presentation to our institution. SSEH is a rare clinical entity with an incidence of approximately 0.1 in 100,000 persons per year [1,2]. This disorder does not have a known cause, but a leading theory supports rupture of the epidural venous plexus as a mechanism of bleeding [3]. It has been associated with a variety of risk factors, including coagulopathy due to primary disease processes or secondary to anticoagulation use [1]. There is no specified treatment strategy for SSEH due to the rarity of the disorder and lack of randomized controlled trials comparing the efficacy of treatment modalities, which include conservative management with medical maximization versus surgical intervention, but most patients undergo surgical intervention due to the perceived risk for persistent or worsening neurological deficit without surgical evacuation of the compressive lesion [5,6]. 

The clinical presentations for both patients described above are consistent with spontaneous epidural hemorrhage in the setting of chronic direct factor Xa inhibition. Direct factor Xa inhibitors are a subgroup of anticoagulant medications considered novel oral anticoagulants. These medications are becoming more commonplace due to their ease of use and efficacy in situations requiring anticoagulation. Some complications regarding use of these direct factor Xa inhibitors are presumably unknown due to their relatively new introduction to clinical practice. Most case reports relating direct factor Xa inhibition to SSEH describe rivaroxaban use [7-11]. To the best of our knowledge this is only the second report of SSEH in the setting of apixaban therapy [4]. Such reports are important in order to raise clinical concern for SSEH in patients on direct factor Xa inhibitor medications who present with acute pain and neurological changes as rapid evaluation and management may be important for maximizing treatment success. 

Our series presents 1 case with intervention less than 12 hours after presentation and another with intervention greater than 12 hours after initial presentation. The first patient experienced a favorable neurological outcome as described above. Unfortunately, the patient described in our second case arrived at our institution in delayed fashion. Although surgical evacuation of the epidural hematoma was successful, the patient did not experience neurological improvement. A previous report describing outcomes for a series of spinal epidural hematoma patients demonstrated the utility of rapid intervention for spinal epidural hematoma of all etiologies (within 12 hours of presentation) in improving neurological outcomes [12]. A more recent study specific to SSEH did not corroborate this finding, but the patient series was smaller and may not have been sufficiently powered [13]. Our experience with the patients described above supports the findings from Lawton et al. that rapid intervention may improve neurological recovery. A factor that may confound our results includes the older age of the patient from case 2. Older age has been related to poor clinical outcome in the setting of SSEH, which may have contributed to the patient’s outcome in addition to the described delay to intervention [1]. Therefore, we are unable to make any conclusions regarding time to intervention from the cases reported here due to the small sample size, the confounding factor of older age, and the observational case report nature of this study. Further study regarding clinical outcomes in relation to time to intervention in SSEH is necessary as data regarding this subject is limited in the literature to this point. 

## Conclusions

SSEH is a rare clinical entity that can result in rapid progression of neurological deficit with risk for permanent deficit. Anticoagulation has been correlated with SSEH via previous case reports and reviews, including the direct factor Xa inhibitor rivaroxaban. However, the cases presented above are among the first reports of SSEH in patients on apixaban therapy, to the best of our knowledge. Further studies regarding treatment strategies and time to intervention will be useful in order to provide high quality care to patients presenting in this clinical scenario. It is important to maintain a high clinical suspicion for SSEH in patients on direct factor Xa inhibitors as time to diagnosis and treatment may have profound effects on neurological outcome. We hope that the above case presentations will assist in raising the clinical suspicion of SSEH in patients presenting with neurological deficit for emergency physicians, internists, neurologists, neurosurgeons, and other practitioners, especially in the setting of anticoagulation with novel oral anticoagulant medications such as apixaban. 
